# Simultaneous and Ultrasensitive Detection of Foodborne Bacteria by Gold Nanoparticles-Amplified Microcantilever Array Biosensor

**DOI:** 10.3389/fchem.2019.00232

**Published:** 2019-04-23

**Authors:** Fengjiao Zheng, Peixi Wang, Qingfeng Du, Yiping Chen, Nan Liu

**Affiliations:** ^1^General Practice Center, Nanhai Hospital, Southern Medical University, Foshan, China; ^2^Department of Clinical Laboratory, The Air Force Hospital of Southern Theater Command of PLA, Guangzhou, China; ^3^College of Food Science and Technology, Huazhong Agricultural University, Wuhan, China

**Keywords:** foodborne pathogen, gold nanoparticle (Au NP), microcantilever array, biosensor, food safety

## Abstract

Foodborne pathogens, especially bacteria, are explicitly threatening public health worldwide. Biosensors represent advances in rapid diagnosis with high sensitivity and selectivity. However, multiplexed analysis and minimal pretreatment are still challenging. We fabricate a gold nanoparticle (Au NP)-amplified microcantilever array biosensor that is capable of determining ultralow concentrations of foodborne bacteria including *Escherichia coli* O157:H7, *Vibrio parahaemolyticus, Salmonella, Staphylococcus aureus, Listeria monocytogenes, Shigella*, etc. The method is much faster than using conventional tools without germiculturing and PCR amplification. The six pairs of ssDNA probes (ssDNA_1_ + ssDNA_2_ partially complementary to the target gene) that originated from the sequence analysis of the specific gene of the bacteria were developed and validated. The ssDNA_1_ probes were modified with -S-(CH_2_)_6_ at the 5′-end and ready to immobilize on the self-assembled monolayers (SAMs) of the sensing cantilevers in the array and couple with Au NPs, while 6-mercapto-1-hexanol SAM modification was carried out on the reference cantilevers to eliminate the interferences by detecting the deflection from the environment induced by non-specific interactions. For multianalyte sensing, the target gene sequence was captured by the ssDNA_2_-Au NPs in the solution, and then the Au NPs-ssDNA_2_-target complex was hybridized with ssNDA_1_ fixed on the beam of the cantilever sensor, which results in a secondary cascade amplification effect. Integrated with the enrichment of the Au NP platform and the microcantilever array sensor detection, multiple bacteria could be rapidly and accurately determined as low as 1–9 cells/mL, and the working ranges were three to four orders of magnitude. There was virtually no cross-reaction among the various probes with different species. As described herein, it holds great potential for rapid, multiplexed, and ultrasensitive detection in food, environment, clinical, and communal samples.

## Introduction

The World Health Organization (WHO) stated that harmful bacteria, viruses, and substances found in unsafe food cause over 200 diseases and an estimated 2 million deaths each year. Foodborne diseases are important causes of morbidity and mortality, and reveal significant impediments to socioeconomic development worldwide. Although most of the foodborne infections are undiagnosed and unreported, annually, 48 million people become ill, 128,000 are hospitalized, and 3,000 die of foodborne diseases. The increasing annual cost of the foodborne illness in the United States alone is estimated at $77.7 billion (Scharff, 2012). For example, as recently as September 2016, *Escherichia coli* (*E. coli*) O157:H7-contaminated beef caused a multistate illness outbreak (http://www.cdc.gov/ecoli/2016/o157h7-09-16/index.html). In August 11, 2017, the deadly *Salmonella* outbreak linked to imported Maradol papayas affected at least 141 people in 19 states, according to the latest update from the Centers for Disease Control and Prevention. *Staphylococcus aureus* (*S. aureus*) is one of the most common human and animal pathogens, is a major cause for concern in multiple infections, and is associated with chronic infections (Liu et al., 2018). *Listeria monocytogenes* (LM) is one type of Gram-positive, non-spore-forming, motile, facultative anaerobic, rod-shaped bacterium that thrives in diverse environments such as water, soil, food products, animals, and humans, and is resistant to freezing, drying, and heat (Hamon et al., 2006). Generally, it is considered as a post-processing contaminant in fully cooked foods.

Culture and colony counting methods are considered as the most classical methods and the most common approaches for verification of the foodborne bacterial pathogens in standardized laboratories; however, they are excessively time-consuming (it might take up to a few days to yield a result) and have limited sensitivity to diverse bacterial pathogens (Lazcka et al., 2007). Currently, real-time or quantitative PCR (Ma et al., 2014; Wang H. et al., 2015) and immunoassay methods (Chattopadhyay et al., 2013; Cho et al., 2014), as well as DNA (Yang et al., 2010; Riahi et al., 2011; Ye et al., 2011), microarrays (Wang et al., 2007; Donhauser et al., 2011; Finetti et al., 2016), and microfluidic platforms (Kim et al., 2014; Zhou et al., 2014; Kim G. et al., 2015; Oh et al., 2016) have increasingly grown. They are employed with relatively fast detection as regards to culture-based methods, yet trained personnel and expensive equipment are also required. Numerous studies have been performed to develop a biosensor to detect these pathogens contained in food (Velusamy et al., 2010; Wang S. et al., 2015; Vaisocherová-Lísalová et al., 2016; Suaifan et al., 2017), which provided high selectivity, label-free protocols and involved minimal sample preparation.

The growing interest in using cantilevers as biosensors is due to their extreme sensitivity and integration. Because of the superior performances of microcantilevers, microcantilever-based biosensors are very attractive to biosensor applications because of their rapid, label-free, real-time, and ultrasensitive features (Jayanthi et al., 2017). It was recently demonstrated that the gene-based cantilever biosensors were competent in measuring DNA hybridization (Johnson and Mutharasan, 2012) *in situ* with continuously flowing liquid samples (Rijal and Mutharasan, 2007). They attract considerable attention because of the abovementioned advantages for detection of target biomolecules. When the specific biomolecular interactions take place between a receptor immobilized on the surface of a cantilever and a target in solution, a mechanical bending of the cantilever arises as a result of a change in surface stress, mass, optical angle, and frequency, which converted biochemical interactions into a concentration-dependent nanomechanical response of the microcantilevers. Applications of these sensors have received great attention ranging from biological sensing (Braun et al., 2009; Kim H. H. et al., 2015; Zhao R. et al., 2015; Chen et al., 2016) and clinical diagnosis (Li et al., 2016) to environmental chemical monitoring (Chen et al., 2015) and pharmacological drug screening (Longo et al., 2013; Huang et al., 2014).

Optical measurements require the use of a beam and a photosensitive detector. Visible light from a diode is shined on the cantilever tip, and the reflected beam moves across the detector surface. The distance between the beams on the detector surface is linked to the degree of deflection (Lang et al., 2002). The optical lever method has been used successfully in the detection of various biochemicals; nevertheless, it suffers from the limitation that the measurements cannot be performed in opaque liquids. By controlling the deflection or changing frequencies of microcantilevers, cantilevers have been developed as a prospective sensing technique with high specificity and sensitivity (Chen et al., 2016). The piezoresistive cantilever sensors measure the changes in electrical conductivity of piezoresistor materials. Unlike the optical method, the piezoresistive method can be used in opaque liquids such as blood or urine. Cantilever sensors have been used successfully in the analysis of genomic material, and the bending of a cantilever caused by intermolecular interactions and conformational changes alters the degree of surface stress (Mukhopadhyay et al., 2005).

Nanomaterials are opening new horizons for fabrication of highly sensitive and specific biosensors especially with their enormous surface-to-volume ratio for highly efficient target interactions. Gold nanoparticles (Au NPs) have been considered as an emerging platform for various biological applications (Sperling et al., 2008) given their good biocompatibility, easy conjugation with biomolecules (He et al., 2018), high surface area, and unique optical properties (Li et al., 2018). Au NP-based DNA biosensors offer a promising platform for the development of rapid, specific, and portable diagnostic strategy for ultra-trace DNA detecting (Wang W. et al., 2015; Han et al., 2017).

**Figure 1:**
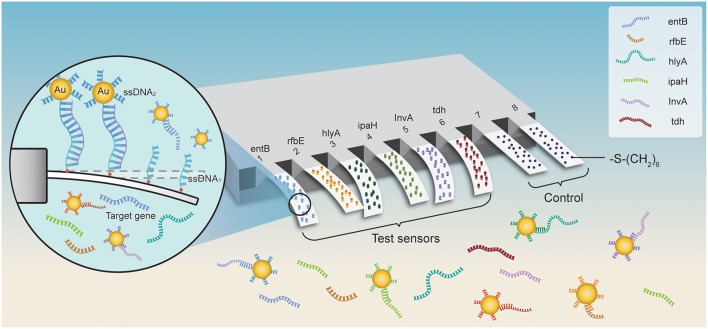
The scheme of a gold-nanoparticle (AU NP)-amplified microcantilever array biosensor for detection of foodborne bacteria. The six pairs of ssDNA probes (ssDNA_1_ + ssDNA_2_ complementary to the target gene) derived from the sequence analysis of the specific gene of the bacteria were developed and validated. The probes of ssDNA_1_ were modified with -S-(CH_2_)_6_ at the 5′-end and ready to immobilize on the self-assembled monolayers (SAMs) of the sensing cantilevers in the array and couple with Au NPs, while the reference cantilevers were modified with 6-mercapto-1-hexanol SAMs to eliminate the interferences from the environment by detecting the deflection induced by non-specific interactions. For multianalyte sensing, the ssDNA_2_ (partial complementary to the target gene) labeling on the Au NPs captured the target gene sequence in the solution, and then the Au NPs-ssDNA_2_-target complex was hybridized with ssNDA1 fixed on the beam of the cantilever sensor resulting in secondary cascade amplification effect. The deflection of microcantilevers was positively correlated with the concentration of the target in the solution.

Herein, we fabricate an Au NP-amplified microcantilever array biosensor that is capable of determining ultralow concentrations of foodborne bacteria including *E. coli* O157:H7, *Vibrio parahaemolyticus* (VP), *Salmonella choleraesuis* (*S. choleraesuis*), *S. aureus*, LM, *Shigella*, etc. The method is much faster than using conventional tools without germiculturing and PCR amplification. The scheme of a Au NP-amplified microcantilever array biosensor for detection of foodborne bacteria is shown in Figure 1. The ssDNA probes are coated on the surface of the microcantilever to make it biosensitive. Then, the modified microcantilever is integrated with Au NPs for signal amplification, and the sensitivity of the microcantilever array biosensor system is greatly improved, which can facilitate the pretreatment process and reduce the cost, thus making the whole process more effective than the other conventional platform. The application is not to be restricted to the field of microbiology, detection of food-contaminated chemicals, diagnosis of clinical biomarkers, or even toxins in hostile environments.

Since the ssDNA_1_ probes interact with targets in the solution, the microcantilevers bending toward the gold side is identified as bending up (positively), and bending toward the opposite side refers to bending down (negatively). By integrating with the enrichment of the Au NP platform and the microcantilever array sensor detection, multiple bacteria can be rapidly and accurately detected at levels as low as 1–9 cfu/ml and the working ranges were three to four orders of magnitude with excellent specificity. The approach can be employed for rapid, multiplexed, and ultrasensitive detection in food, environment, clinical, and communal samples.

## Materials and Methods

### Chemicals and Materials

Mutanolysin (3,000 U/mL), tris(2-carboxyethyl)phosphine hydrochloride (TCEP, ≥98%), hydrogen tetrachloroaurate gold (HAuCl_4_), sodium citrate, DL-dithiothreitol (DTT), sodium dodecyl sulfate (SDS), mercaptoundecanoic acid (MUA) ethanol, and 6-mercapto-1-hexanol (MCH) were purchased from Sigma-Aldrich (St. Louis, MO, USA). Phosphate-buffered saline (PBS, pH 7.4) served as the binding and washing buffer. All the ssDNA probes were synthesized by Sangon (Shanghai, China) and dissolved in the immobilization buffer (10 mmol/L PBS containing 100 μmol/L TCEP, pH 7.4). Teflon tubes with an inner diameter of 0.8 mm and an outer diameter of 0.9 mm were made by Tianjin Scientific Apparatus Company (Tianjin, China). Ultra-purified water was prepared by an ultrapure water production system, Milli-Q system (Millipore, Bedford, MA; minimum resistivity, 18.2 MΩ cm^−1^). All the experiments were performed and maintained at room temperature (RT, 25 ± 2°C).

### Pre-preparation of the Foodborne Bacteria

The microorganisms used in this study were obtained from the American Type Cell Collection (ATCC) and the National Center for Medical Culture Collections of China (CMCC), which are listed in Table S1. The LM strains used in this experiment were obtained from the Institute of Microorganism in Beijing. All the other strains were cultured and maintained in 500 ml of nutrient broth at 28°C with shaking for 48 h, harvested by centrifugation (5,000 × g, 20 min), and then stored in 50 ml of sterilized PBS (10 mM phosphate and 150 mM NaCl). For the next experiment, PBS solution was used to dilute the bacteria's concentration in a gradient. After revival through a conventional method, each strain of these foodborne bacteria was inoculated on its corresponding medium and incubated for 6 h.

### Preparation of the ssDNA Probes for Capturing the Foodborne Bacteria

The specific gene sequences of these target bacteria were downloaded from Genbank (https://blast.ncbi.nlm.nih.gov/Blast.cgi). These ssDNA probes have a base pair number of 25, which are partially complementary sequences of the target genes of the selected foodborne bacteria. Probe 1 ssDNAs were modified with -SH-(CH)_6_ at the 5′-end and readily to immobilize on the cantilevers (ssDNA_1_s). Probe 2 ssDNAs were also modified with -S-(CH_2_)_6_ at the 5′-end (ssDNA_2_s) and ready to couple with Au NPs. The sequences of these two groups of ssDNAs are complementary to the 3′ and 5′ ends of the target DNAs. All of these abovementioned sequences including probes and target genes were synthesized by Sangon Biotechnology Co., Ltd (Shanghai, China) and listed in Table 1.

**Table 1:** Sequences of the DNA probes used for the detection of the foodborne bacteria.

**Bacteria**	**ssDNA_1_ (5^′^-3^′^)**	**ssDNA_2_ (5^′^-3^′^)**	**Target gene**	**Length (bp)**
*E. coli* O157:H7	CCCAATTGACAATACAACATGACGA	AAAATGCTCACCCCGCCACCTTAAC	*rfb*E	1,287
VP	TTTGGCATTACATTTTTCTTTTGCC	TAAATAACTTTTATTGCCGTTATGA	*tdh*	223
*S. choleraesuis*	CACGACGAAAGAGATGAATTGTCAC	TAAATGAATTTCTTCACGAGTCTGT	*Inv*A	1,568
*S. aureus*	CCTTATTGTTTTGTACTTCCTTTGG	TTAAATAACTTTTATTGCCGTTATGA	*ent*B	246
LM	CGTTTTAAACCATGCCGTAAATTTC	TCGAACCCTTACCACCTCTTGCCAA	*hly*A	831
*Shigella dysenteriae*	GAGTGTACCTTGTTAGAGGCCTTTT	ACCAGTCTTCGGCACCTTCTCTTACT	*ipa*H	320

### Immobilization of DNA Probe on the Microcantilever Array

Microfabricated arrays consist of eight silicon cantilevers that are 500 μm long, 90 μm wide, and 1 μm thick, and 20-nm-thick gold on one side is obtained from Micromotive GmbH, Mainz, Germany. The microcantilever was cleaned and washed in Piranha solution (98% H_2_SO_4_ in 30% H_2_O_2_, 7:3 v:v) for 10 min, immersed in 30% NH_3_ (5 min), rinsed twice with dH_2_O, and dried in N_2_ before use. After careful cleaning, the six testing interdigital electrodes in the array were functionalized with 100 nM ssDNA_1_ probes by inserting them into Teflon tubes filled with immobilization buffer for 3 h at RT to serve as the sensing microcantilevers. As a result, we blocked all the active sites on the other two remaining microcantilever electrodes by MCH to work as reference microcantilevers to prevent non-specific adsorptions. After blocking by MCH, the interdigital electrodes of the microcantilever array were carefully and respectively, inserted into short Teflon tubes and the measurement cell of the equipment and fixed on a holder. The other end of the Teflon tube was sealed off. The mechanical response of the microcantilever was continuously compared with the excitation wave by a network analyzer with a sweep time of 1 s Hewlett Packard, 4589A to record the amplitude and the phase spectrum. To increase the sensitivity, higher modes (modes 10–15) were selected for measurement.

### Synthesis of the Au Nanoparticles and the Coupling of the ssDNA_2_ to Au Nanoparticles

All the glassware used in the experiments were soaked and cleaned in the freshly prepared 3:1 HNO_3_-HCl solution for 24 h, rinsed thoroughly in pure water, and dried in air before use. Colloidal Au NPs were synthesized by sodium borohydride and sodium citrate reductions according to our published protocols (Zhou et al., 2011). The procedure is briefly described as follows: 100 mL of 0.01% (w/w) HAuCl_4_ solution, 1 mL of 1% trisodium citrate, and 1 mL of 0.075% NaBH_4_-1% trisodium citrate solution were mixed under vigorous stirring at RT for 30 min for Au NP preparation. Then, another 1 mL of 0.02% NaN_3_ was added into the obtained Au NP solution for antisepsis and stabilization. The obtained solution was filtered by 0.22-μm filters and then stored at 4°C. The sizes of the Au NPs were then examined by transmission electron microscopy (TEM).

The synthesized Au NPs were modified by the following procedures: ~33 μg of thiol-DNA probe was mixed with 200 μl of 0.1 MDT in total darkness for ~3 h to obtain the reduced thiol-DNA probe. This solution was then passed through a Nap-5 column for purification of the reduced thiol-DNA probe. The purified DNA probe was then mixed with 1 mL of Au NP solution, and the obtained mixture was kept in the dark overnight. PBS with pH 7.0 was added to the above mixture to a final concentration of 9 mM phosphate. Then, SDS was added to the mixture to a final concentration of 0.1% (w/v), and the mixture was stirred for 30 min. Afterwards, NaCl solution was added to the above solution six times over 2 days until reaching a final concentration of 0.3 M. After the addition of NaCl, the solution was repeatedly centrifuged at 11,000 × g for 5 min and resuspended with 200 μl of 100 mM PBS three times to separate excess unreacted ssDNA from the thiol-ssDNA-Au NP product. Following the final washing, the thiol-ssDNA-Au pellet was resuspended in 0.5 ml of 0.01% SDS solution and then it was characterized by UV–visible spectroscopy and stored at 4°C before usage.

### Measurement Procedure

Ten microliters of 100 nM ssDNA_2_-Au NPs was employed as the partial complement of the target. The probe and the target ssDNA (100 μl) with a series of gradient concentrations were stored and vortexed at 800 rpm in 1 mM PBS (pH 7.4) for 1 h at RT. The running buffer was flowed through the functionalized interdigital electrodes of the microcantilever array and then pumped to the waste reservoir via syringe pump (Standard PHD ULTRA™ CP, Harvard Apparatus, USA) at a flow rate of 0.40 μl/s during the experiment. The microcantilever array was equilibrated until a stable baseline was obtained under the running buffer for ~10 min. Six interdigital electrodes of the cantilever were performed as detection group and the other two electrodes of the cantilever were placed parallel as control. The change in the degree of deflection is monitored as an electrical signal by putting the piezoresistor in a Wheatstone bridge structure, and the voltage was 3.0 V.

### Sensitivity and Specificity for the Foodborne Bacteria of the Microcantilever Array Biosensor

**Figure 2:**
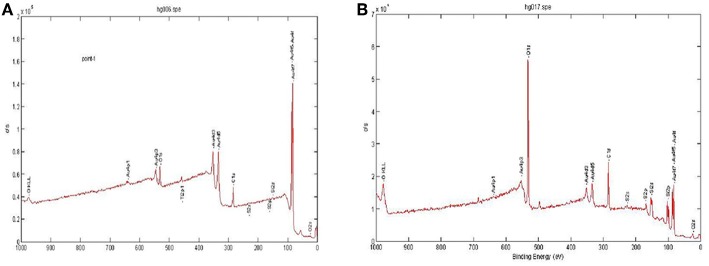
X-ray photoelectron spectroscopy (XPS) analysis of the microcantilever with or without HS-ssDNA oligomer SAM configuration. **(A)** Details of the XPS analysis of the microcantilever with SAM configuration. **(B)** Details of the XPS analysis of the reference microcantilever without SAM configuration.

The genomic DNA (gDNA) of the experimental bacterial extraction was adapted from the literature (Wilson, 2013) with some modifications. All the listed strains of the bacterial cells were centrifuged at 12,000 × g for 10 min. After that, the supernatant was discarded, and the sediment, i.e., the bacteria's cell pellet at the bottom of the inner wall of the EP tube, was resuspended in 200 μl of 1 M Tris–HCl (pH 8.0) for thrice repeated resuspension. Then, 30 μl of 10% SDS and 15 μl of proteinase K were added in the resuspended solution and incubated for 1 h at 37°C. Sequentially, 100 μl of 5 mol/L NaCl was blended with 80 μl of cetyl trimethyl ammonium bromide (CTAB)/NaCl solution in the tube and incubated for 10 min at 65°C for DNA extraction and purification of the bacteria. Afterwards, chloroform/isopentanol (24:1, v:v) with equal volume was mixed well and centrifuged at 8,000 × g for 4–5 min and then the supernatant was transferred into a new EP tube. Isopropanol with 0.8 times the volume of the obtained supernatant was slowly added and gently vortexed (600 rpm) until the flocculated precipitate (extracted DNA) appeared. The obtained precipitate could be centrifuged at 4,000 × g for 4–5 min and washed by 1 ml of 70% ethanol. Finally, it was dissolved in 200 μl of TE buffer (RNaseA < 25 ng/ml) and stored at −20°C. One milliliter of target DNA with a series of gradient concentrations (Figure 2) in EP tubes was boiled at 95°C for 3 min to obtain the target ssDNAs, and then the tubes were quickly placed in a mixture of ice water to prevent DNA renaturation. The lowest detection limit (LDL) was determined.

### Preparation of Milk Samples

Pasteurized whole milk samples were acquired from the local supermarket with Tetra-Pak™ aseptic packaging in Guangzhou, China. Different volumes of the six experimental strains of foodborne bacteria were spiked in 10 tubes of 10 ml of milk as unknown samples. The last sample (No. 10) was designated as the blank control. To avoid the non-specific absorption and interference, all milk samples were pretreated via our previously used method by trichloroacetic acid for quick removal of proteins and grease in milk products (Zhou et al., 2011).

## Results and Discussion

### The Selection for the Specific Genes of the Foodborne Bacteria

The specific genes in these selected foodborne bacteria are listed in Table 1. It has already been proven that the *rfbE* gene is specific to this serotype of *E. coli* O157:H7, and the antigen being expressed by this gene is closely related to severe clinical symptoms (Abdulmawjood et al., 2004). We also select the *invA* and *tdh* genes coding the invasion protein of *Salmonella* as the most used specific gene for identification of *Salmonella* (Malorny et al., 2003) and VP. The *tdh* gene is considered to be strongly associated with the clinical strains by molecular epidemiological studies. It has been reported that more than 95% of VP was isolated from US patients with *diarrhea*-carried *tdh* gene (DePaola et al., 2003). The *hlyA* gene that codes for the listeriolysin O toxin has been used. The *hlyA* gene (Gene Accession No. X 12157) is 1,717 base pairs (bp) long, and only a single copy of this gene is revealed in the genome of pathogenic LMs. As one of the specific genes in *Shigella*, it has been verified that there are multiple *ipaH* genes in all *Shigella* genomes (Yang et al., 2005).

### Validation of Immobilization of ssDNA Probe on the Microcantilever

To confirm the immobilization of the DNA probe, the X-ray photoelectron spectroscopy (XPS) measurement was employed to evaluate the self-assembly MUA immobilized layer onto the gold surface. XPS is uniquely suited for the analysis of such a thin layer onto the microcantilever. When the sample is subjected to high-energy X-rays, the fine elements on the surface with ~2–10 nm can be measured by the initiating photoelectron energy distribution. Details of the XPS analysis are demonstrated in Figure 2. For the microcantilever with the Au layer, a very strong Au peak was observed in Figure 2A, whereas a low Au peak and a strong O peak were observed in the reference microcantilever shown in Figure 2B. The strong O peak revealed the basic composition of the microcantilever, i.e., silicon dioxide. These results suggested that the self-assembled monolayer (SAM) was successfully sputtered on the surface of the piezoresistive microcantilever.

The SAM acts as an active platform for the immobilization of thiol-modified oligonucleotides. Semiconductor sensing elements can be characterized by large coefficients and have therefore been applied extensively in the measurement of pressure, strain, and flow. The piezoresistive effect is rather small in metal but much more pronounced with regard to semiconductors. Once the target analyte makes contact with the SAM, the probe can capture it and a surface stress variation of the microcantilever surface occurs. The variation generates deflection and stress in the microcantilever whose measurement determines the targets' type and concentration. Silicon-based microcantilever sensors have been developed using pn-junction isolated piezoresistors for stabling the voltage. This layer is embedded in a Wheatstone bridge, which is a vital structure that is responsible for transforming the change in the degree of deflection into an electrical signal. Due to the identical mechanical structure, it has a relatively better suppression and counteraction on the mechanical and external environmental noise (Sharma and Mutharasan, 2013). Fluorescence microscopy was employed for the direct observation of the microcantilever whether the fluorescence-labeled ssDNA has been immobilized on it with 492-nm exciting light. We found that no fluorescent signal could be observed in the unmodified piezoresistive microcantilever shown in Figure S1A, and uniformly dark yellow green fluorescence is shown in Figure S1B. It could directly prove ssDNA immobilization on the microcantilever via the cross-linking effect of EDC and NHS.

### Sensitivity and Selectivity of the Au Nanoparticle-Amplified Microcantilever Array Biosensor

After introduction of the target gene in the solution, the functionalized ssDNA probe (ssDNA_1_) is immobilized on the sensing interdigital electrodes of the microcantilevers and the partial complementary to the target gene ssDNA_2_-Au NPs hybridized to the target gene. Then, the binding of ssDNA-target-Au NP caused a significant change in the surface stress of the electrodes of the microcantilevers, forcing them to bend to the silicon side. As a consequence, defection (Δ*X*) differences between the sensing and reference cantilevers were produced. The deflection of microcantilevers was positively correlated with the concentration of the target in the solution.

**Figure 3:**
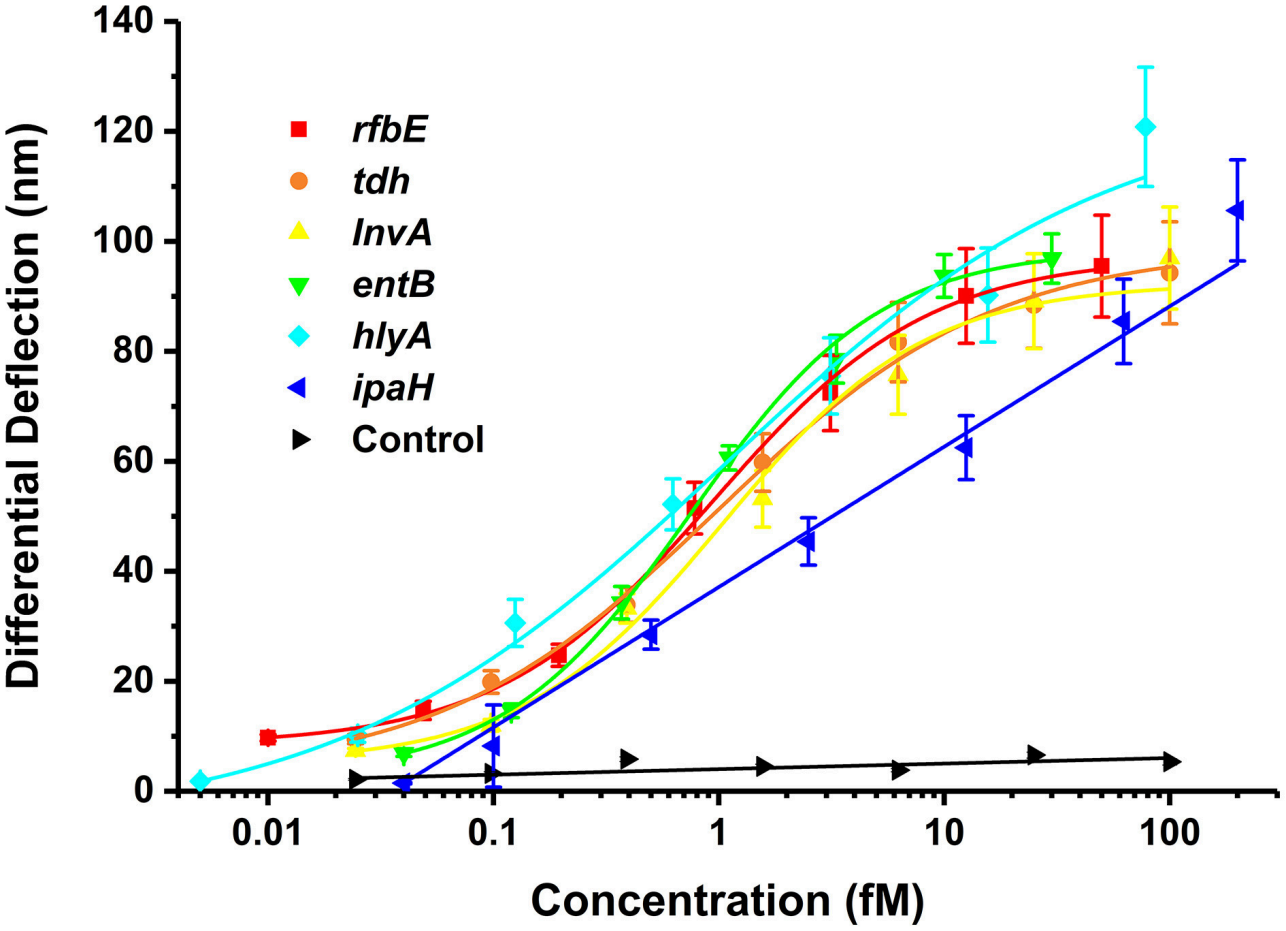
Relationship between differential deflection (nm) and the concentration (fM) of the specific genes of foodborne bacteria at series of gradient concentrations (*n* = 3).

By optimizing the working conditions and investigating various target concentration gradients, the logistic regression standard curves of the multiplex analysis of microcantilever biosensor were well-plotted (Figure 3). Intense electrical signals were observed when the target DNA was exposed to the probe of the sensor. Furthermore, when the concentration of the target DNA increased, the output voltage increased dramatically. The signal was logistically related to the concentration of target DNA. The sensitivity of the piezoresistive is determined by the change in electrical resistance of the piezoresistor and the origin of the surface stress applied to the surface of the microcantilever. The coefficients of determination *R*^2^ for all the gene targets (ranging from 0.990 to 0.999) demonstrated a good correlation in the 6-plex assay and detection ranges were from 3 to 4 log units. The minimum detectable concentrations were 0.005–0.040 fM shown in Table 2. The LDLs were 1.824–15.385 aM, which were defined by 3σ (where σ is the standard deviation of the blank solution). As measured in counting number equivalents, the LDLs were 1–9 cells/ml. Owing to the small sample volume in this assay, the LDLs are exhibited by the absolute number of bacteria instead of the commonly used bacterial concentrations, i.e., the colony forming unit without amplification of the bacteria's colonies. It demonstrated that the sensor used throughout this experiment is sufficiently sensitive to effectively detect the bacteria's target DNA.

**Table 2:** Standard curves for targets' genes of the foodborne bacteria and the related determination parameters.

**Bacteria**	**Target gene**	**Standard curve**	**Working range (fM)**	**Working range (cells/ml)**	**LDL (aM)**	**LDL (cells/ml)**	***R*^**2**^**
*E. coli* O157:H7	*rfb*E	*Y* = 97.334 – 88.983/[1 + (*x*/0.944)^0.907^]	0.010–50.000	6–3 × 10^4^	3.254	2	0.998
VP	*tdh*	*Y* = 98.880 – 95.910/[1 + (*x*/0.978)^0.706^]	0.024–100.000	14–6 × 10^4^	6.463	4	0.997
*S. choleraesuis*	*Inv*A	*Y* = 92.358 – 87.301/[1 + (*x*/1.040)^0.972^]	0.024–100.000	14–6 × 10^4^	6.865	4	0.990
*S. aureus*	*ent*B	*Y* = 98.679 – 96.163/[1 + (*x*/0.752)^1.039^]	0.040–30.000	24–1.667 × 10^4^	8.696	5	0.999
LM	*hly*A	*Y* = 125.300 – 131.828/[1 + (*x*/1.057)^0.503^]	0.005–78.125	3–4.688 × 10^4^	1.824	1	0.995
*S. dysenteriae*	*ipa*H	*Y* = 37.155 + 25.526*x*	0.040–200.000	24–1.2 × 10^5^	15.385	9	0.997

**Figure 4:**
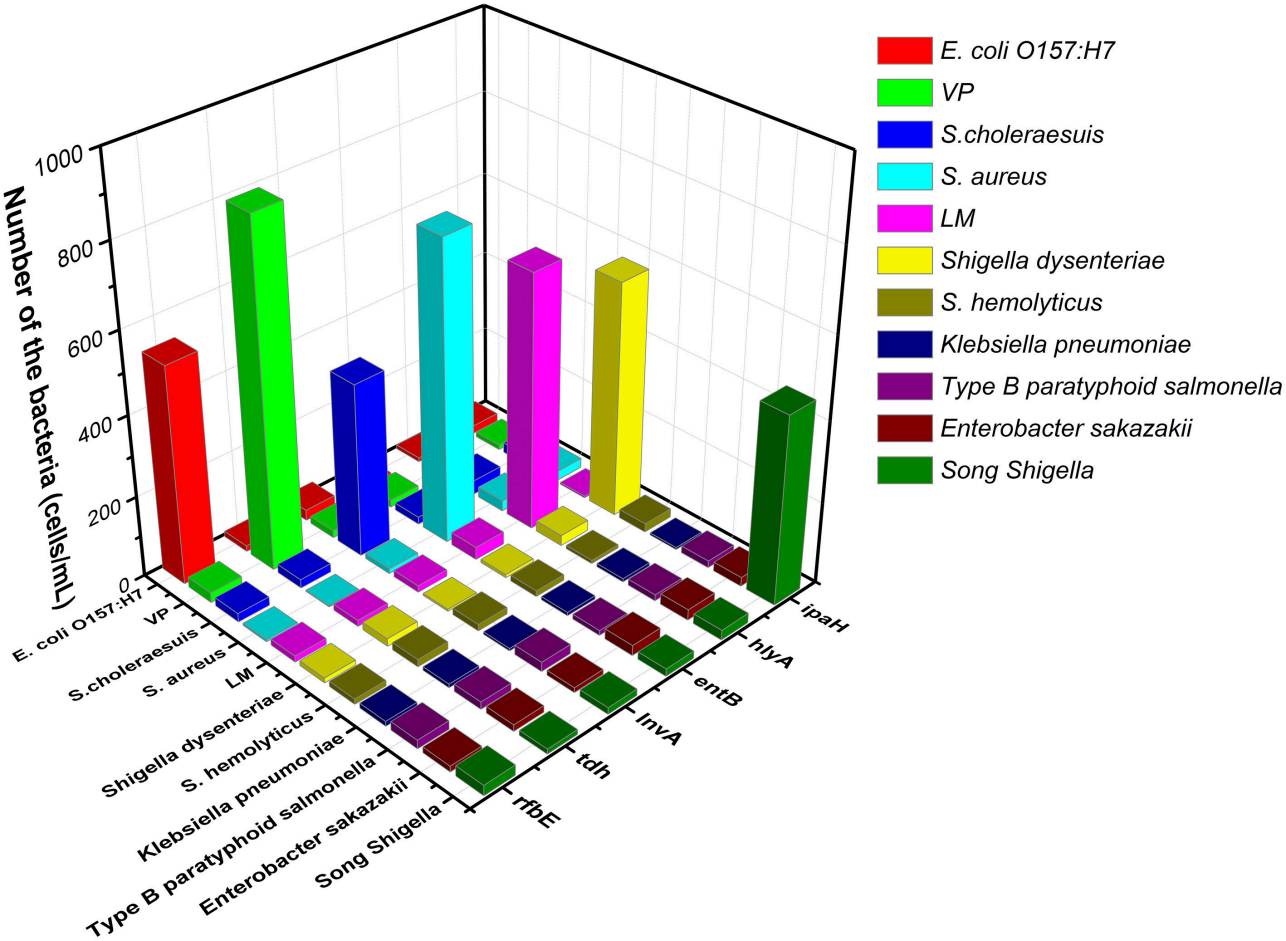
The selectivity of the microcantilever array biosensor for detecting of the specific genes of foodborne bacteria.

For specificity of the Au NP-amplified microcantilever array biosensor, the target strains and other foodborne strains were separately tested by using the proposed piezoresistive microcantilever biosensor under the same condition. Based on the species specificity of the probe for the bacteria, the specific gene belongs to the same species, which can lead to cross-reaction, such as *Shigella dysenteriae* and *Song Shigella*. The electrical signals were observed to be significantly increased upon the addition of the treated solution of the target strains and were greater than those of the other strain groups, and we did not find an obvious change in Δ*X* upon exposure to various concentrations of other genes as shown in Figure 4. Based on the obtained results of the specificity, detection of the microcantilever array biosensor is dependent on the specificity of DNA probes. Another reason lies on the formation of helix, which prevents the non-specific adsorption onto the surface of Au NP (Chen et al., 2009). Specific microbial genes are essential in designing highly specific probes for the testing of a specific microorganism, which is consistent with a previous study (Sun et al., 2014). It suggested that the Au NP-amplified microcantilever array biosensor exhibited good selectivity for detecting foodborne bacteria with different species.

### Effect of Modification With Au Nanoparticles

**Figure 5:**
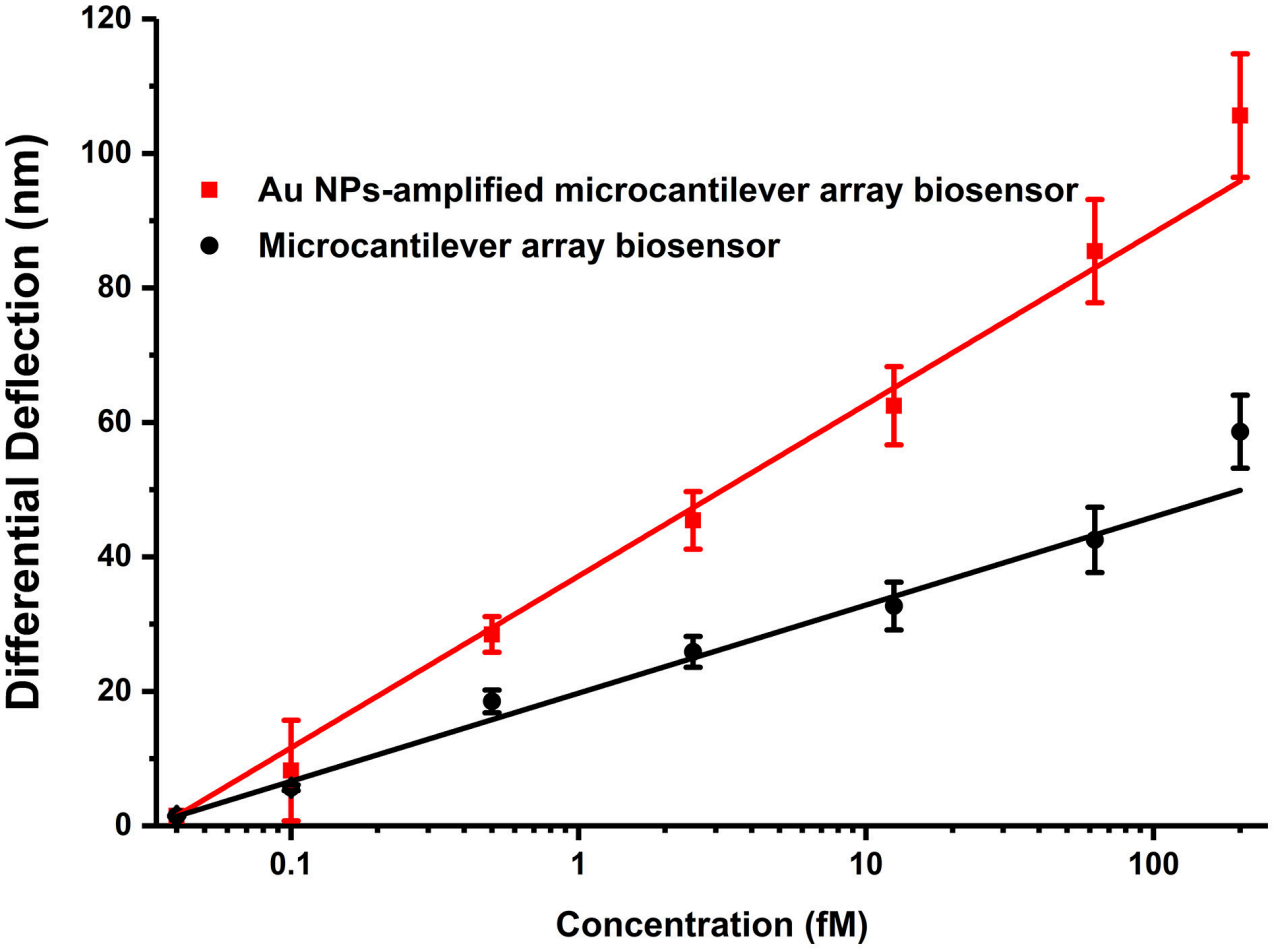
Effect of modification by Au NPs.

The above self-assembly procedure provides a versatile and convenient technology for the DNAs of foodborne bacteria detection. The particle sizes of Au NPs prepared using the sodium borohydride method were ~5 nm, as determined by TEM (Figure S2). Au NPs have been previously used to amplify signals in surface plasmon resonance and quartz crystal microbalance analysis by improving the mass of bio-samples to increase the sensitivity of biosensors (He et al., 2014). Au NP-labeled ssDNA_2_ hybridizing to the part of the target gene would increase the mass of the sensor only if the target strand genes appear on the sensor (Sharma and Mutharasan, 2013). The two types of probes, i.e., the thiol-DNA probe and the thiol-Au NP-DNA probe, were used under totally identical reaction conditions. It is an effective method to improve the amount of loaded molecules of ssDNA probes and significantly enhance the signal, i.e., improve the sensitivity of the whole analysis by Au NPs (Figure 5).

The structures of the biological molecules reacting with each other in the surface of the microcantilever were changed and then led to the change of the cantilever's piezoresistive dimensions and deflection. DNA hybridization immobilized on Au NP with competent spatial freedom has distinctly greater thermal stability than the pure double strand (Akamatsu et al., 2006). When binding to the Au NPs, the DNA probes can be more stable (Zhao X. et al., 2015) and exhibit rapid kinetics and high efficiency for sequence-specific hybridization (Yao et al., 2015). Furthermore, this approach has the potential of achieving sensitivity comparable to pre-enrichment LDL of PCR assays. The carbon chain structure, i.e., -(CH_2_)_6_-, which is chemically modified on the ssDNA_1_ probe, allows it to extend into space where the reducing steric hindrance and is favorable for hybridization reaction.

### Detection of the Foodborne Bacteria in Milk Samples

**Figure 6:**
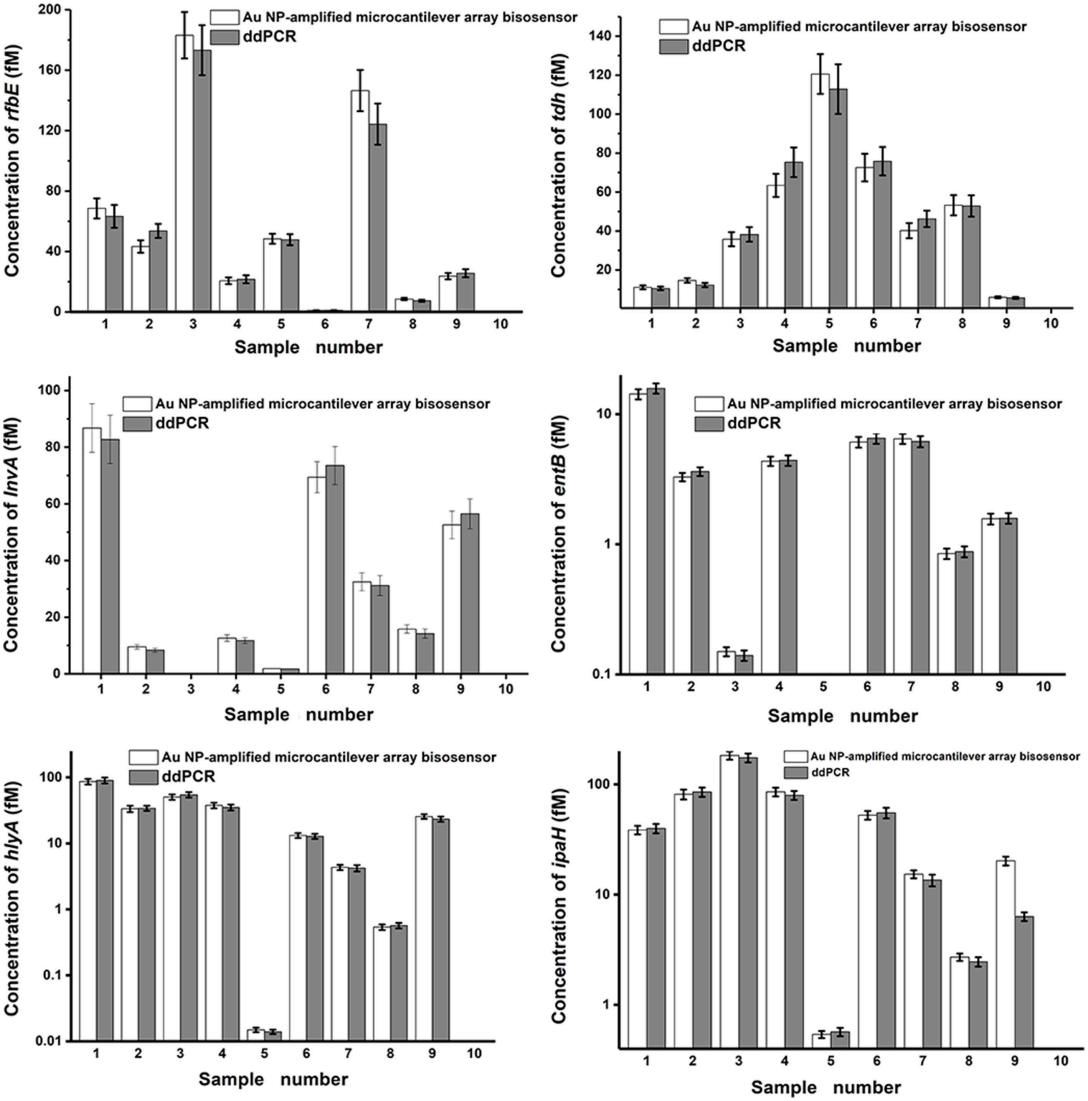
Detection of the specific genes of foodborne bacteria in milk samples. Nos. 1–9 were the unknown milk samples and No. 10 was the blank control sample.

To further investigate the reliability and accuracy of the Au NP-amplified microcantilever array biosensor, we used the proposed method to analyze 10 spiked foodborne bacteria in milk samples. The procedures also could be employed for detecting the foodborne bacteria in milk samples, which were extracted by a boiling method. As droplet digital PCR (ddPCR) is a relatively novel and commonly accepted method that enables the absolute quantification of a single molecule of nucleic acid (Huang et al., 2015), it was employed to detect real milk samples to confirm feasibility in comparison with the Au NP-amplified microcantilever array biosensor. Experimental results showed that these two methods were in agreement with each other (Figure 6, *P* > 0.05). The obtained CVs of the Au NP-amplified microcantilever array biosensor were below 9.97%, which suggests an eligible accuracy and reproducibility within 10%. These results show that the developed method was practical for the detection of foodborne bacteria in milk samples. The whole process could be accomplished in <1 h.

Table 3 shows the reported biosensor technologies, including oligonucleotide microarray (Wang et al., 2007), chemiluminescence (CL) flow-through microarray (Donhauser et al., 2011), suspension array technology (Sun et al., 2014), microparticle enhanced dsDNA probes (Riahi et al., 2011), and integrated lab-on-a-disc (Kim et al., 2014; Oh et al., 2016) for detection of foodborne bacteria. Compared with the reported technologies, the detection parameters of our proposed work, such as working range, detection time, and LDL have considerably improved. Although it has no advantage on the throughput capacity compared with microarray technology, the accuracy and the cost can be controlled, and the modification and detection process are relatively simple.

**Table 3:** Summary of biosensor technologies for detection of multiple foodborne bacteria.

**Method**	**Description**	**Single/multiple detection (*n*)**	**Target bacteria**	**Working range**	**Detection time (h)**	**LDL**	**References**
Oligonucleotide microarray	A high-throughput detection and identification system that uses universal PCR primers to amplify a variable region of the bacterial 16S rRNA gene, followed by reverse hybridization of the products to species-specific oligonucleotide probes on a chip.	Multiple (204)	Pure culture belonging to 13 genera of bacteria and 115 strands were isolated from foods	10^2^-10^6^ cfu/ml	<4	10^1^-10^2^ cfu/ml	Wang et al., 2007
CL flow-through DNA microarray assay	Using the stopped PCR strategy, the amount of amplified target DNA is strongly dependent on the applied cell concentration. The generation of single-stranded DNA sequences is essential for DNA hybridization assays on microarrays.	Multiple (3)	*E. coli* O157:H7, *Salmonella enterica*, and *Campylobacter jejuni*	100–10^4^, 10–10^4^, and 1–100 cells/ml, respectively	3.5	136, 500, and 1 cell/ml, respectively	Donhauser et al., 2011
Suspension array technology	The assay uses a liquid suspension hybridization format with specific oligonucleotide probes covalently bound to the surface of fluorescent color-coded microspheres.	Multiple (6)	*E. coli* O157:H7, *Shigella, Salmonella*, VP, *S. aureus* and LM	1–1 × 10^8^ cfu/ml	>14	20–4 × 10^3^ cfu/ml	Sun et al., 2014
Microparticle-enhanced dsDNA probes	A microparticle enhanced double-stranded DNA probe is demonstrated for rapid species-specific detection of bacterial 16S rRNA.	Single	*S. saprophyticus, Enterococcus* spp., and *P. mirabilis* one by one	2 × 10^4^-2 × 10^1^ cfu/ml	<1	8 cfu/ml	Riahi et al., 2011
Integrated Lab-on-a-disc	A centrifugal microfluidic device, which integrated the three main steps of pathogen detection, DNA extraction, isothermal recombinase polymerase amplification (RPA), and detection, onto a single disc is developed.	Single	*Salmonella*	10^1^-10^6^ cfu/ml in PBS and 10^2^-10^6^ cfu/ml in milk	0.5	10–100 cfu/ml	Kim et al., 2014
Integrated Lab-on-a-disc	All the processes including DNA extraction and purification, DNA amplification, and amplicon detection were integrated on a single disc. Silica microbeads incorporated in the disc enabled extraction and purification of bacterial genomic DNA from bacteria-contaminated milk samples.	Multiple (10)	10 strains including *E. coli* O157:H7, *Salmonella typhimurium*, VP and LM, etc.	10^2^-10^4^ cells/ml	1	1–10 cells/ml	Oh et al., 2016
Au NP-amplified microcantilever array biosensor	Integrated with the enrichment of the Au NP platform and the microcantilever array sensor detection.	Multiple (6)	*E. coli* O157:H7, VP, *Salmonella, S. aureus*, LM, and *Shigella*	3–1.2 × 10^5^ cells/ml	<1	1–9 cells/ml	This work

*cfu, colony forming unit*.

## Conclusion

In this experiment, we have designed a piezoresistive microcantilever biosensor for the simultaneous and ultrasensitive detection of foodborne bacteria by Au NP amplification. The established biosensor has advantages such as rapid detection (~1 h), excellent specificity, and high sensitivity without germiculturing and PCR amplification, which is superior to conventional tools. Therefore, this biosensor could be a promising alternative for further applications in food safety, environment, and the clinical field as a point-of-care diagnostic tool.

## Author Contributions

NL, QD, and YC contributed to the experimental design, data analysis and interpretation, manuscript writing, and manuscript revision. FZ and PW contributed to the material synthesis and characterizations, and data acquisition.

### Conflict of Interest Statement

The authors declare that the research was conducted in the absence of any commercial or financial relationships that could be construed as a potential conflict of interest.
