# Supplementary material for: Simultaneous and Ultrasensitive Detection of Foodborne Bacteria by Gold Nanoparticles-Amplified Microcantilever Array Biosensor
Source: Front Chem. 2019 Apr 23;7:232. doi: 10.3389/fchem.2019.00232 (PMC6489696; doi:10.3389/fchem.2019.00232)
Supplement: Supplementary file 1 [file Data_Sheet_1.docx]

**Supplementary Material**

Simultaneous and Ultra-sensitive Detection of Foodborne Bacteria by Gold Nanoparticles-amplified Microcantilever Array Biosensor

Fengjiao Zheng^1, 2, †^, Peixi Wang^1, †^, Qingfeng Du^1*^, Yiping Chen^3*^, Nan Liu^1*^

1. General Practice Center, Nanhai Hospital, Southern Medical University, Foshan, 528244, P. R. China

2. Department of Clinical Laboratory, The 458th Hospital of PLA, Guangzhou, 510602, P. R. China

3. College of Food Science and Technology, Huazhong Agricultural University, Wuhan, 430070, P. R. China

*Correspondence:

Nan Liu

nhyy8132@sum.edu.cn

orcid.org/0000-0002-8895-3169

Qingfeng Du

nhyyqk@126.com

Yiping Chen

[chenyiping@mail.hzau.edu.cn](mailto:chenyiping@mail.hzau.edu.cn)

^†^ These authors have contributed equally to this work.

**Supplementary Tables**

**Table S1** The foodborne bacteria used in the experiment

| **Bacterial strain** | **Resource** |
| --- | --- |
| [*Escherichia*](javascript:void(0);) [*coli*](javascript:void(0);) O157:H7 | ATCC 25922 |
| *Vibrio parahaemolyticus* | ATCC 17802 |
| *Salmonella* *choleraesuis* | ATCC 13312 |
| *Staphylococcus aureus* | ATCC 26075 |
| *Listeria monocytogenes* | ATCC 13932 |
| *Shigella dysenteriae* | CMCC 51252 |
| *Streptococcus hemolyticus* | CMCC 32210 |
| *Klebsiella pneumoniae* | CMCC 46117 |
| *Yersinia enterocolitis* | CMCC 52225 |
| *Type b paratyphoid salmonella* | CMCC 50094 |
| *Enterobacter sakazakii* | CMCC 45401 |
| *Song Shigella* | CMCC51592 |


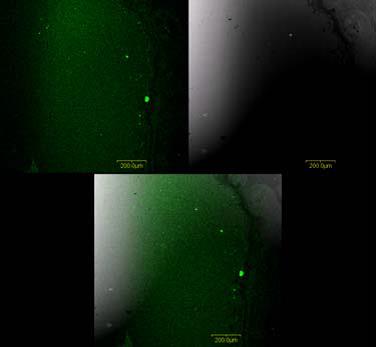
**Supplementary Figures**


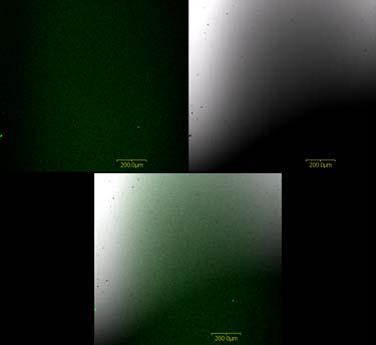


1. (B)

**Fig. S1.** The fluorescence microscopic image of piezoresistive microcantilever.

1. The unmodified piezoresistive microcantilever; (B) the modified piezoresistive microcantilever by ssDNA probe.


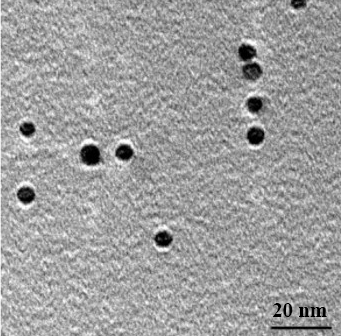


**Figure S2.** TEM images of the prepared Au NPs by sodium borohydride reduction method.
